# Construction of an Artificial Vagina from Small Intestine

**Published:** 1912-10

**Authors:** 


Construction of an Artificial Vagina from Small Intestine.—In *Weiner Med. Wochensch.* No. 49, Dr. Halban is reported to have constructed an artificial vagina from small intestine. After performing laparotomy he incised the peritoneal fold between the bladder and the rectum and then separated the bladder from the rectum as far up as the vaginal *cul de sac.* He then excluded a section of the small intestine and united the central and peripheral portions of the gut by lateral anastomosis. Then he implanted the excluded intestinal segment into the vulva and sutured the incised peritoneal fold leaving an opening for the passage of the mesentery of the implanted segment of gut. Lastly he enlarged the vaginal opening and sutured the gut into position. The operation proved a complete success from a functional point of view.

